# Supplementary material for: Low-temperature paddlewheel effect in glassy solid electrolytes
Source: Nat Commun. 2020 Mar 20;11:1483. doi: 10.1038/s41467-020-15245-5 (PMC7083903; doi:10.1038/s41467-020-15245-5)
Supplement: Supplementary file 1 — Supplementary Information [file 41467_2020_15245_MOESM1_ESM.pdf]

Supplementary Information

Low-Temperature Paddlewheel Effect in Glassy Solid Electrolytes

Smith et al.

Supplementary Figures

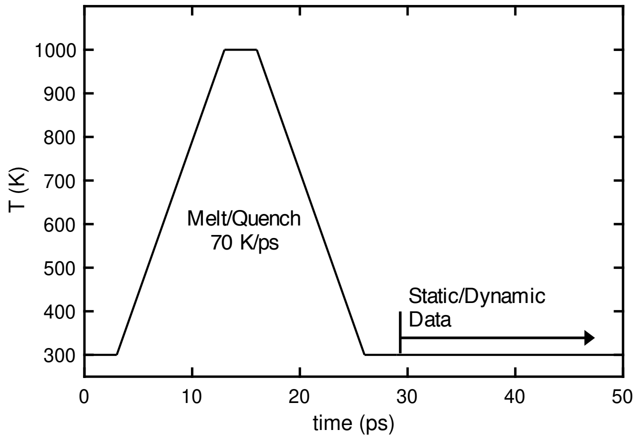

Supplementary Figure 1. *Ab initio* molecular dynamics (AIMD) melt-and-quench procedure for generating models of glassy Li_3PS_4. This procedure was applied using the NPT ensemble at four different pressures, resulting in 4 glass models with different densities, as shown in Supplementary Table 1.

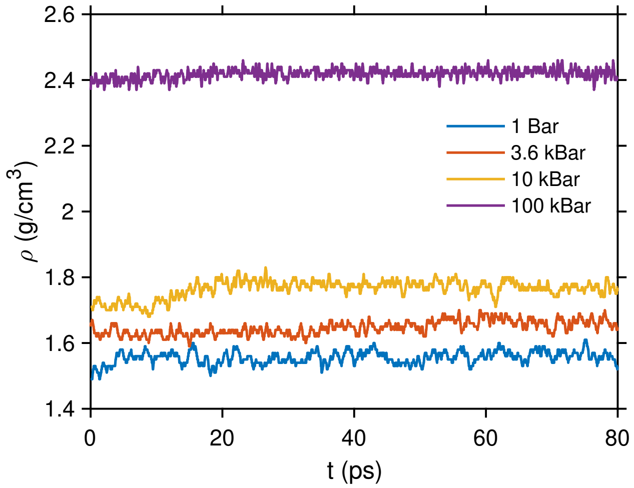

Supplementary Figure 2. Density of glassy $75Li_2S$-$25P_2S_5$ as a function of hydrostatic pressure at 300 K over an 80 ps AIMD trajectory. The initial equilibration phase of duration 3 ps is not shown. The calculated density for the glass and crystalline γ-Li_3PS_4 averaged over 80 ps is shown in Supplementary Table 1. The experimental density of $75Li_2S$-$25P_2S_5$ glasses prepared at room temperature with a pressure of 1.8 and 3.6 kBar were reported as 1.45 and 1.68 g cm^{-3}, respectively.[1] Baba and Kawamura calculated an AIMD density of 1.79 g cm^{-3} using a computational cell that was constrained to be cubic.[2] For comparison, the experimental density of γ-Li_3PS_4 (1.93 g cm^{-3})[3] is also shown in Supplementary Table 1.

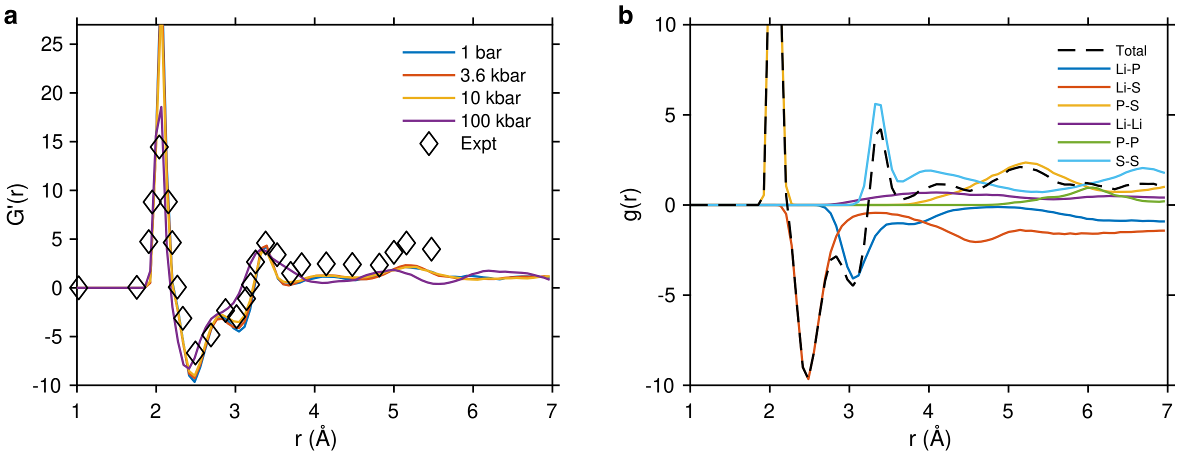

Supplementary Figure 3. Neutron pair distribution function data. (a) Calculated total neutron pair distribution function for 4 glassy LPS models generated at T = 300 K and with different hydrostatic pressures (solid lines) compared to experimental data (diamonds) from Ohara *et al.*[4] (b) Calculated total and partial neutron pair distribution function at T = 300 K and 1 bar.

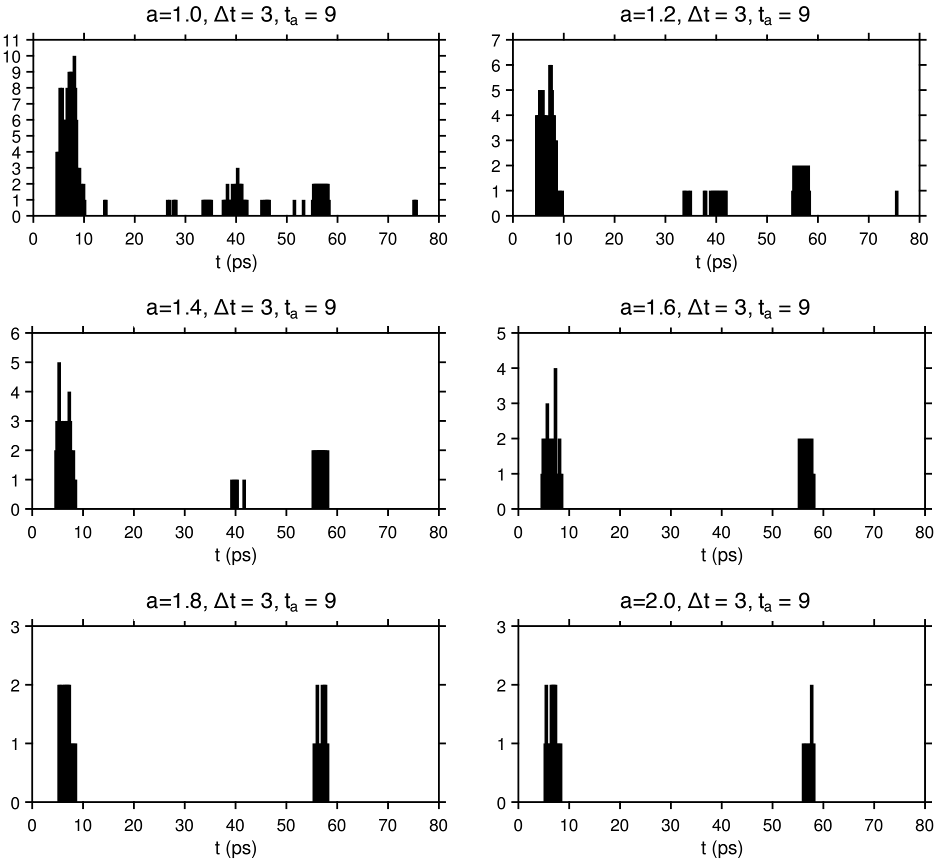

Supplementary Figure 4. Effect of varying the Li-ion displacement threshold, a (Equation 3, main text), on the detection of correlated ion migration events in LPS glass at 300 K and 1 bar. The value of a is varied from 1 to 2 Å while keeping the parameters $\Delta t = 3$ ps and $t_a = 9$ ps constant. Here, Δt is the residence time that both precedes and follows a displacement event, and t_a is the time window that includes the residence times and transition time.

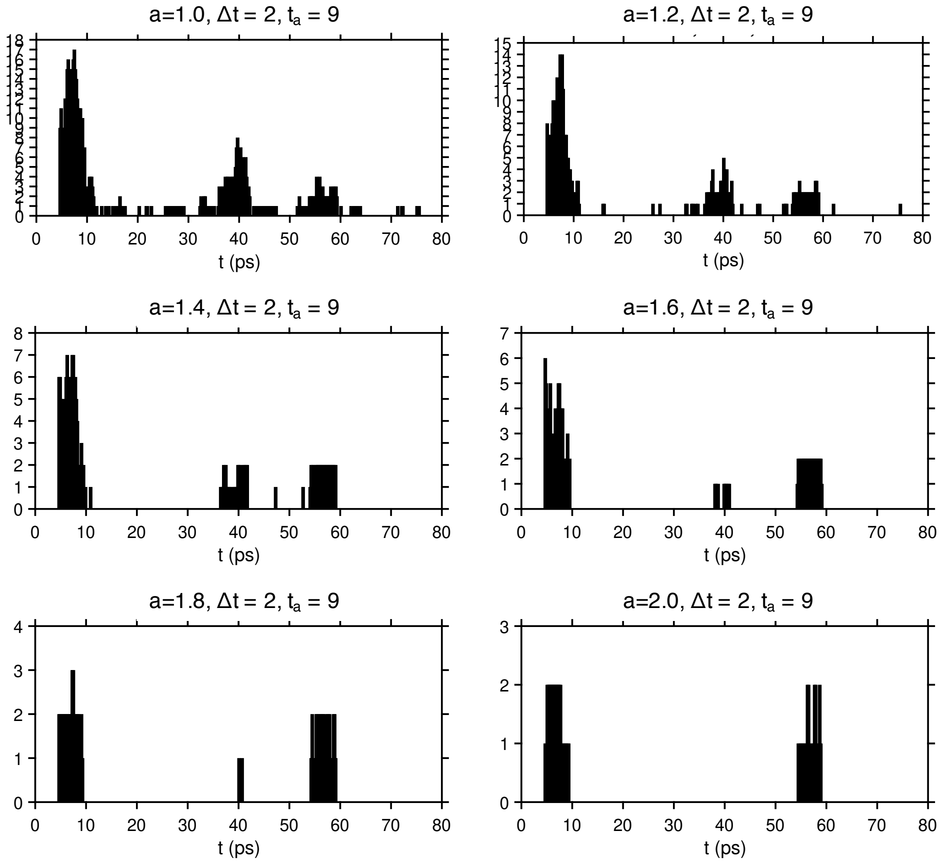

Supplementary Figure 5. Effect of varying the Li-ion displacement threshold, *a* (Equation 3, main text), on the detection of correlated ion migration events in LPS glass at 300 K and 1 bar. The value of *a* is varied from 1 to 2 Å, while keeping the parameters $\Delta t = 2$ ps and $t_a = 9$ ps constant. Here, Δt is the residence time that both precedes and follows a displacement event, and t_a is the time window that includes the residence times and transition time.

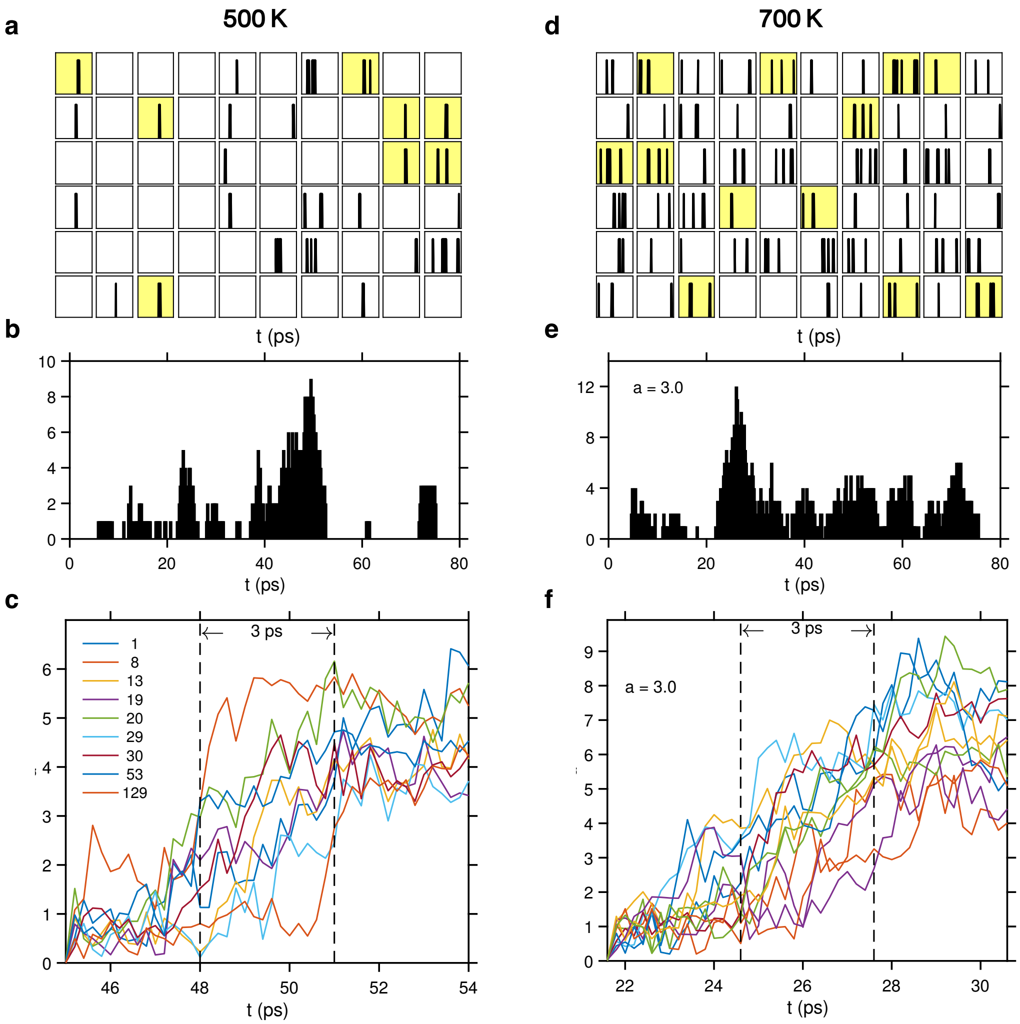

Supplementary Figure 6. Identification of individual and cooperative Li-ion migration events (Equation 3, main text) at 500 K (left column) and 1 bar, and at 700 K (right column) and 1 bar. The value of *a* is set to 1.6 Å at 500 K and to 3.0 Å at 700 K; otherwise both temperatures use the same parameter values for Δt = 3 ps and t_a = 9 ps. (a,d) Individual Li-ion migration events. (b,e) Number of atoms participating in a cooperative migration event as a function of the time which those events were observed. The yellow shading in (a,d) identify the Li-ions that participate in the cooperative event involving the largest number of ions in panel (b) at 49 ps and at 25 ps in panel (e). (c,f) Displacements of the cooperatively-migrating ions identified in (a,b) and (d,e) relative to the beginning of their residence time.

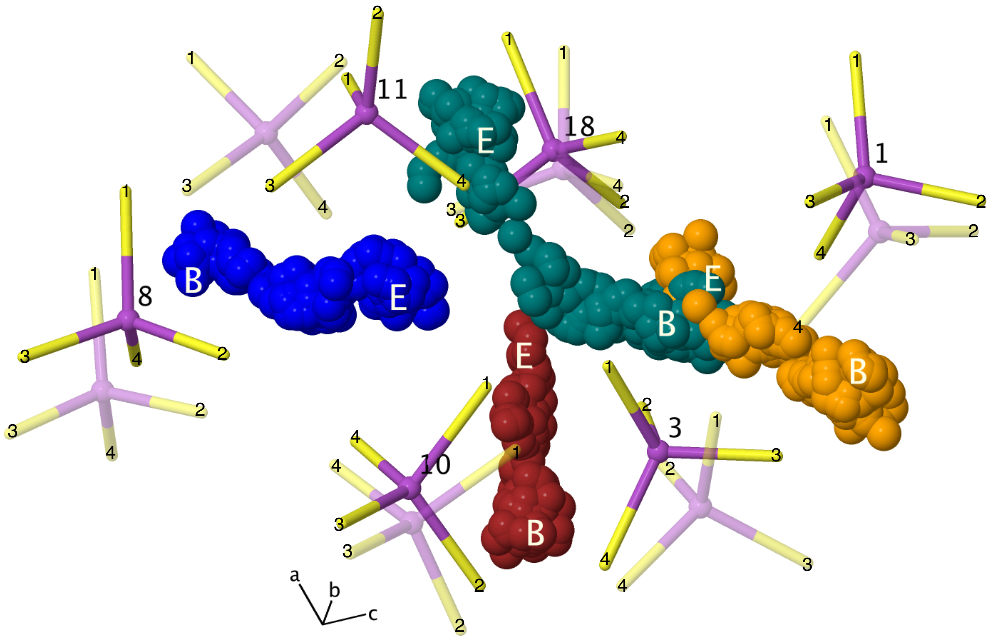

Supplementary Figure 7. Alternative view of Fig. 4 (main text) showing cation-anion cooperative motion involving several adjacent cations and anion reorientations at 300 K. Distinct colored spheres represent four different lithium ions superimposed at 40 fs intervals over a 10 ps trajectory; initial and final positions of these ions are labeled "B" and "E", respectively. Tetrahedral PS_4 anions are colored magenta (phosphorus) and yellow (sulfur). The initial positions of the anions at the start of the migration event are shown with partial transparency; opaque depictions indicate their final positions. Large numeric labels identify individual anions discussed in the main text; small numerals identify the and initial and final positions of sulfur atoms. For clarity, only a portion of the simulation cell is shown. The local temporal and spatial correlations of migrating cations and neighboring anions were analyzed over 640 ps of trajectories. The displacement of individual lithium and angular displacements of three nearest neighbor anions are shown in Supplementary Figures 8-16. Overall, the data indicate migrating lithium is correlated with the rotational motion of neighboring anions at low temperatures.

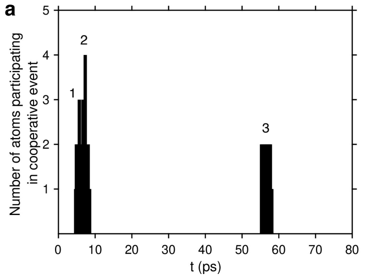

b

Event	Time	ID1	ID2	ID3	ID4
1	5.7	3	49	50	
2	7.3	3	19	50	52
3	56.7	15	37		

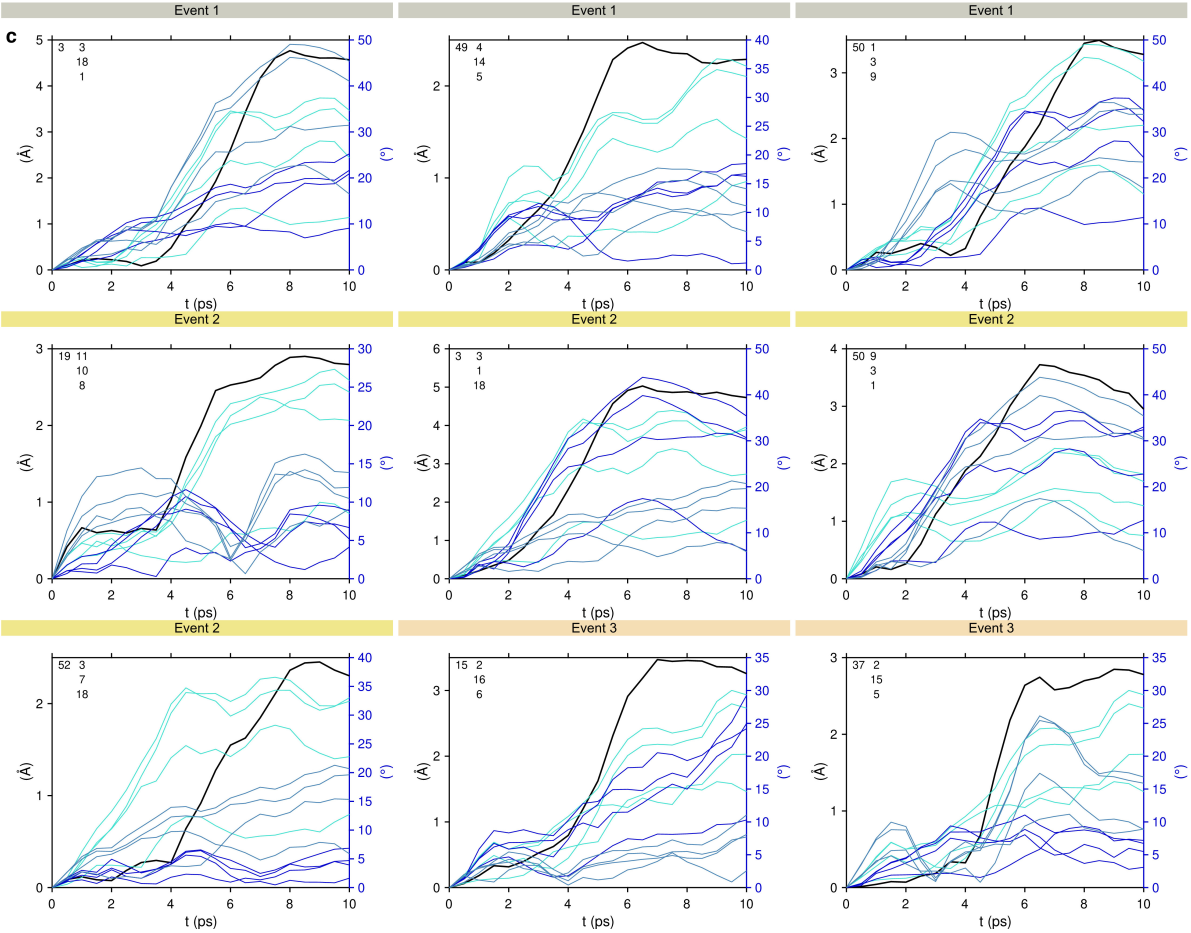

Supplementary Figure 8. Characterization of Li-ion migration events, and their correlation with rotational displacements of PS_4^{3-} anions, in LPS glass at 300 K (MD run #1). (a) Identification of migration events using Eq. 3 in the main text. Numbers located above the histogram bars identify events whose displacements were examined for paddlewheel dynamics. (b) Table summarizing the simulation time at which each event identified in (a) occurred, and the identification (ID) number of the displaced atoms. Lithium ions have IDs that range from 1 to 60; Sulfur ions have IDs ranging from 81 to 160. (c) Displacements of Li-ions (black line, left axis) that participate in the migration events identified in (a-b), and the coinciding angular displacements of the three nearest-neighbor PS_4 anions (blue hues, right axis). The ID for each Li and PS_4 anions appear in the top left corner of each plot. Four rotational displacements are plotted for each anion using a common color; these displacements correspond to rotations of the 4 sulfur atoms about the center of mass of the PS_4 tetrahedra. To more clearly identify long-lived Li displacements (and to distinguish them from shorter vibrational excursions), Li and PS_4 angular positions in panel (c) are averaged over a moving time window of width equal to 2 ps.

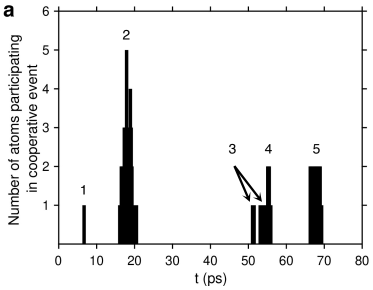

b

Event	Time	ID1	ID2	ID3	ID4	ID5
1	6.7	37				
2	17.9	1	29	39	44	116
3	51.1	57				
3	51.5	19				
4	55.3	17	47			
5	68.1	17	47			

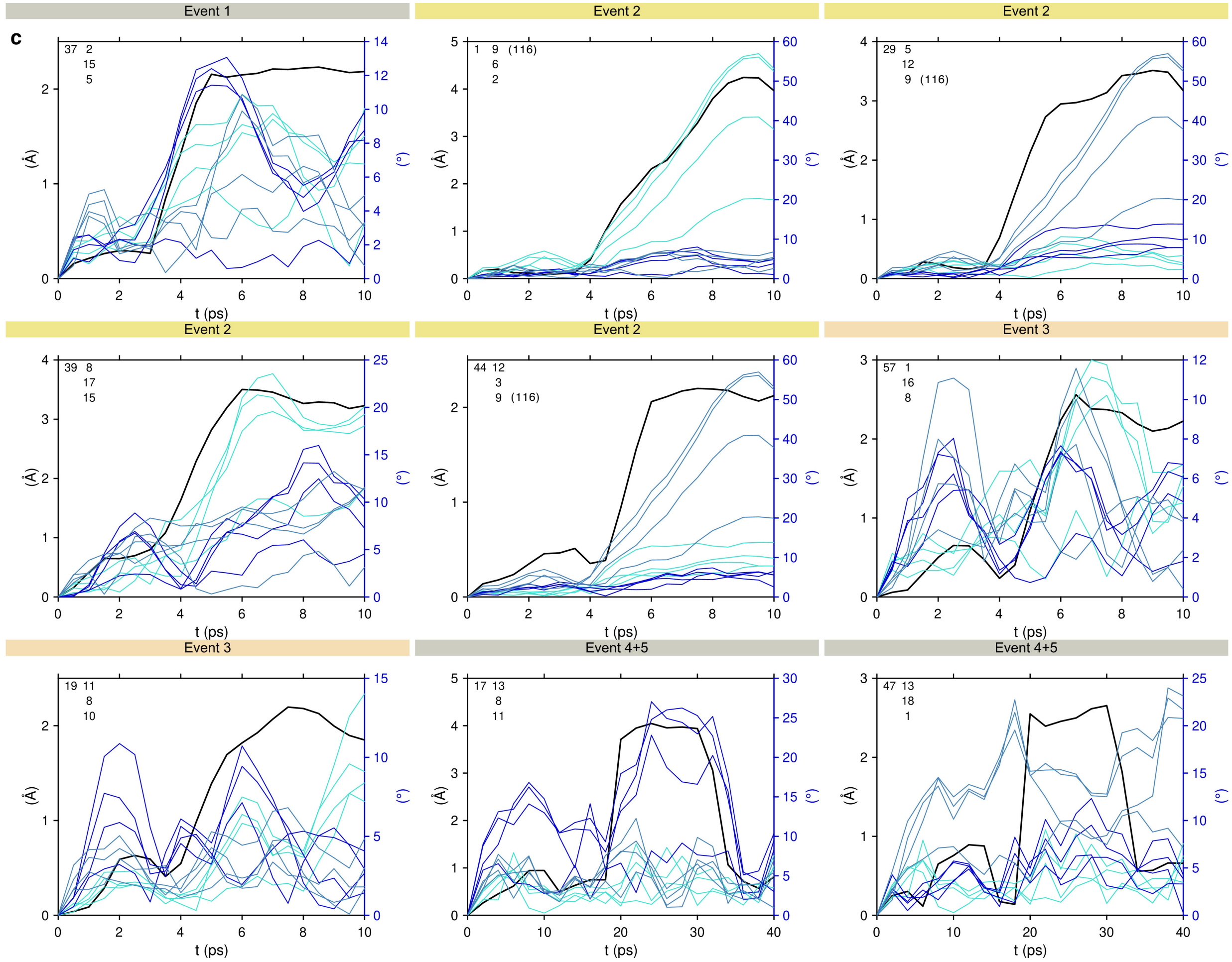

Supplementary Figure 9. Characterization of Li-ion migration events, and their correlation with rotational displacements of PS_4^{3-} anions, in LPS glass at 300 K (MD run #2). See the caption of Supplementary Fig. 8 for additional details.

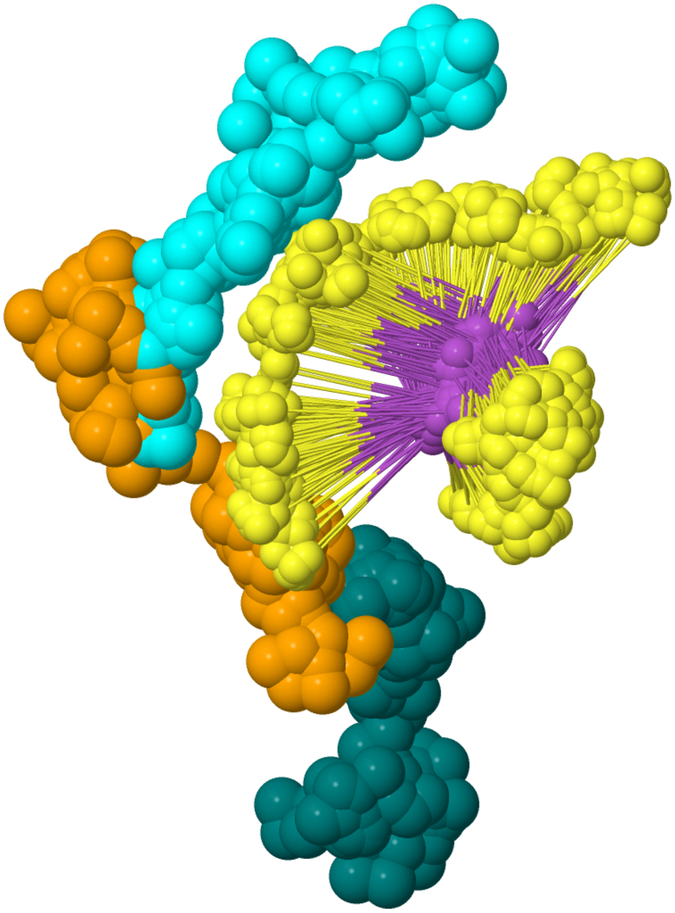

Supplementary Figure 10. Real-space trajectory of Event 2 in Supplementary Figure 9. Migration of Li-ions, 1-cyan, 29-orange, 44-teal are locally correlated with the rotation of anion 9.

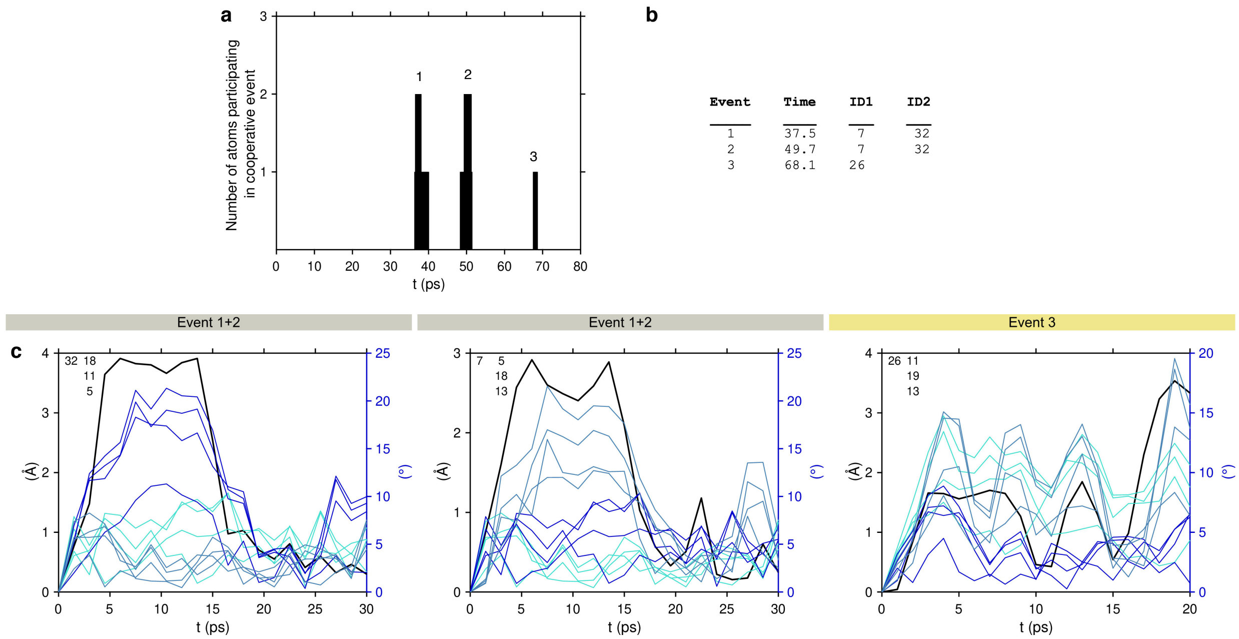

Supplementary Figure 11. Characterization of Li-ion migration events, and their correlation with rotational displacements of PS_4^{3-} anions, in LPS glass at 300 K (MD run #3). See the caption of Supplementary Fig. 8 for additional details.

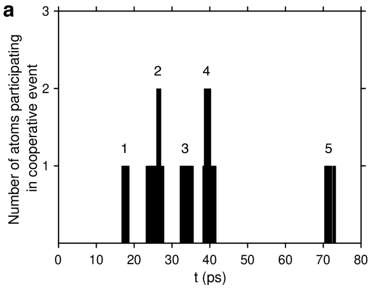

b

Event	Time	ID1	ID2
1	17.7	52	
2	26.3	19	110
3	33.9	40	
4	39.7	7	13
5	71.3	52	

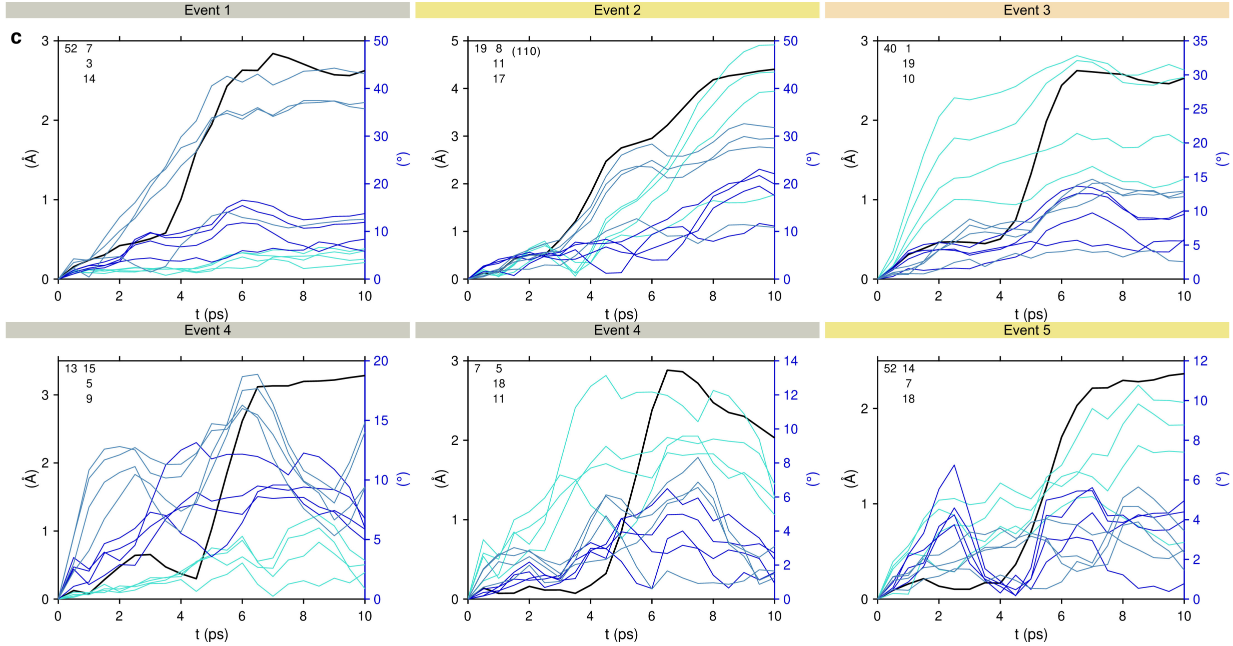

Supplementary Figure 12. Characterization of Li-ion migration events, and their correlation with rotational displacements of PS_4^{3-} anions, in LPS glass at 300 K (MD run #4). See the caption of Supplementary Fig. 8 for additional details.

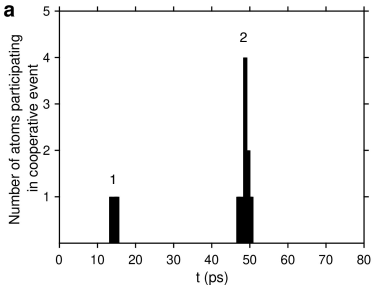

b

Event	Time	ID1	ID2	ID3	ID4
1	14.5	14			
2	48.7	14	32	39	59

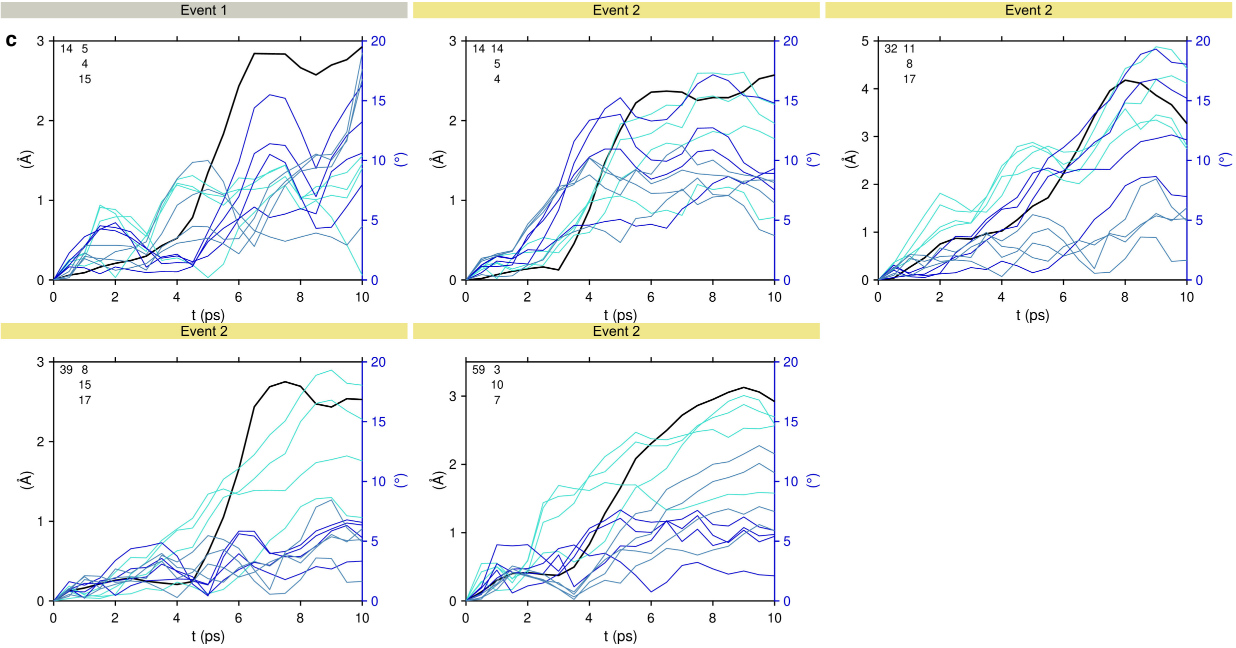

Supplementary Figure 13. Characterization of Li-ion migration events, and their correlation with rotational displacements of PS_4^{3-} anions, in LPS glass at 300 K (MD run #5). See the caption of Supplementary Fig. 8 for additional details.

a

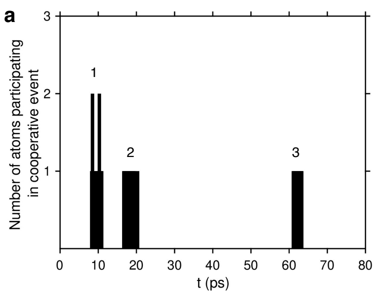

b

Event	Time	ID1	ID2
1	8.5	8	30
1	10.3	8	30
2	17.9	8	
3	62.1	13	

c

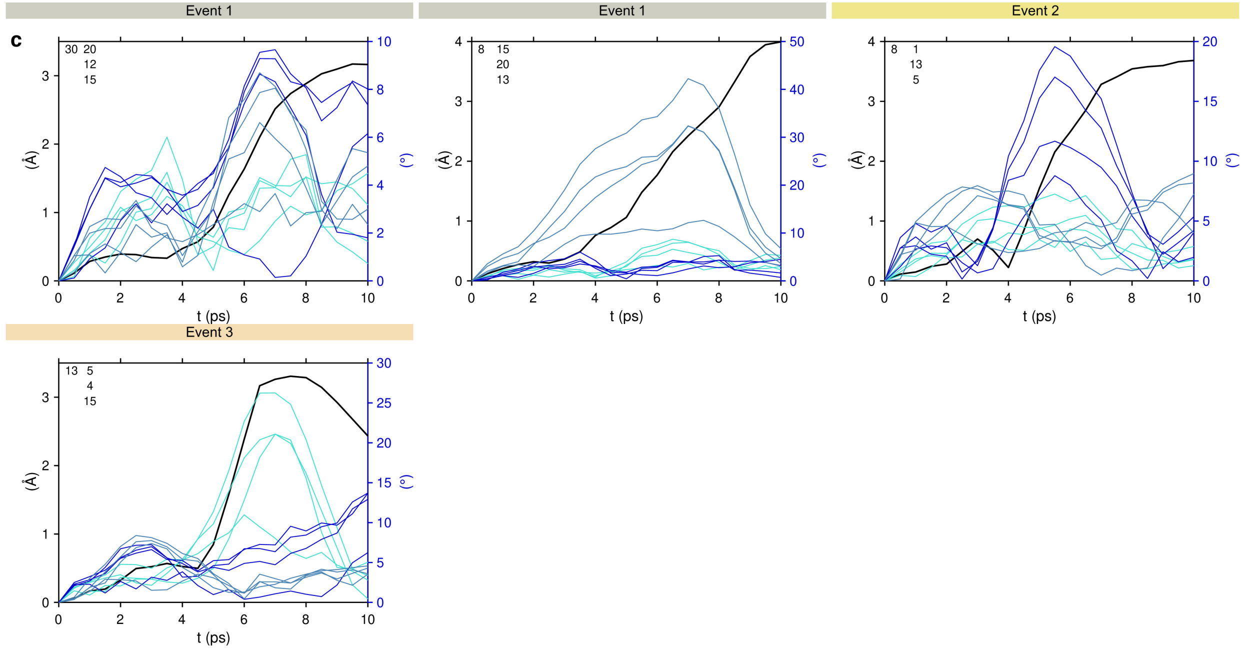

Supplementary Figure 14. Characterization of Li-ion migration events, and their correlation with rotational displacements of PS_4^{3-} anions, in LPS glass at 300 K (MD run #6). See the caption of Supplementary Fig. 8 for additional details.

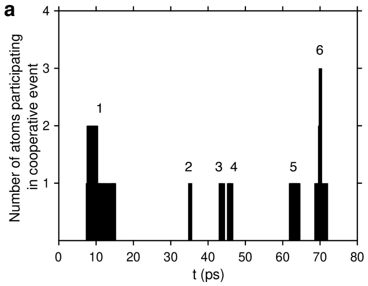

b

Event	Time	ID1	ID2	ID3
1	9.1	133	135	
1	12.3	21		
1	12.7	46		
1	13.5	50		
1	14.5	37		
2	35.1	12		
3	43.5	41		
4	45.9	4		
5	64.3	21		
6	69.9	38	44	
6	70.1	38	107	108

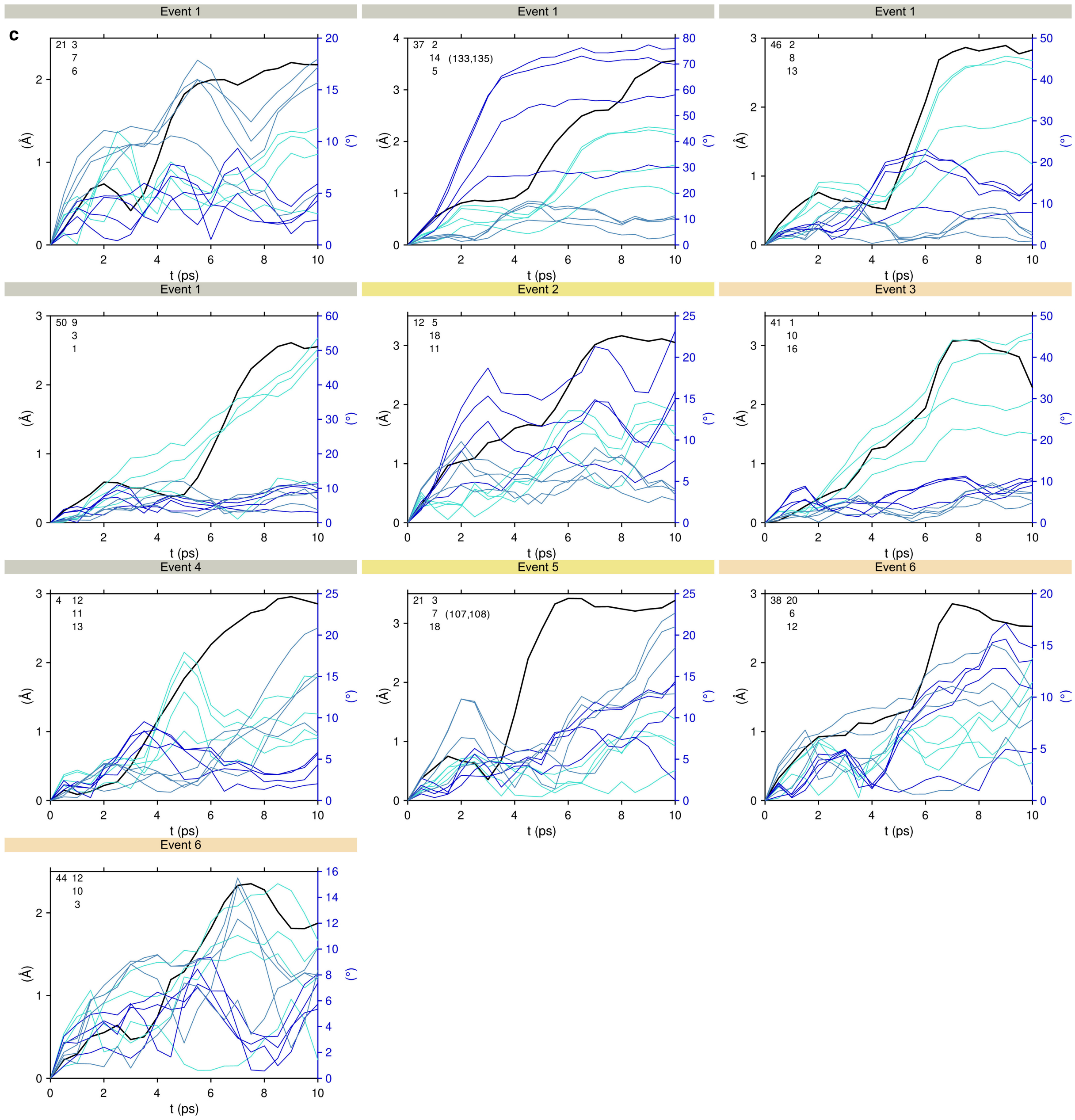

Supplementary Figure 15. Characterization of Li-ion migration events, and their correlation with rotational displacements of PS_4^{3-} anions, in LPS glass at 400 K (MD run #7). See the caption of Supplementary Fig. 8 for additional details.

a

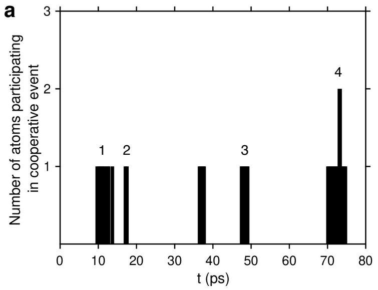

b

Event	Time	ID1	ID2
1	10.7	38	
1	12.7	41	
2	17.1	38	
	37.1	106	
3	48.1	1	
4	71.1	59	
4	73.1	21	44

c

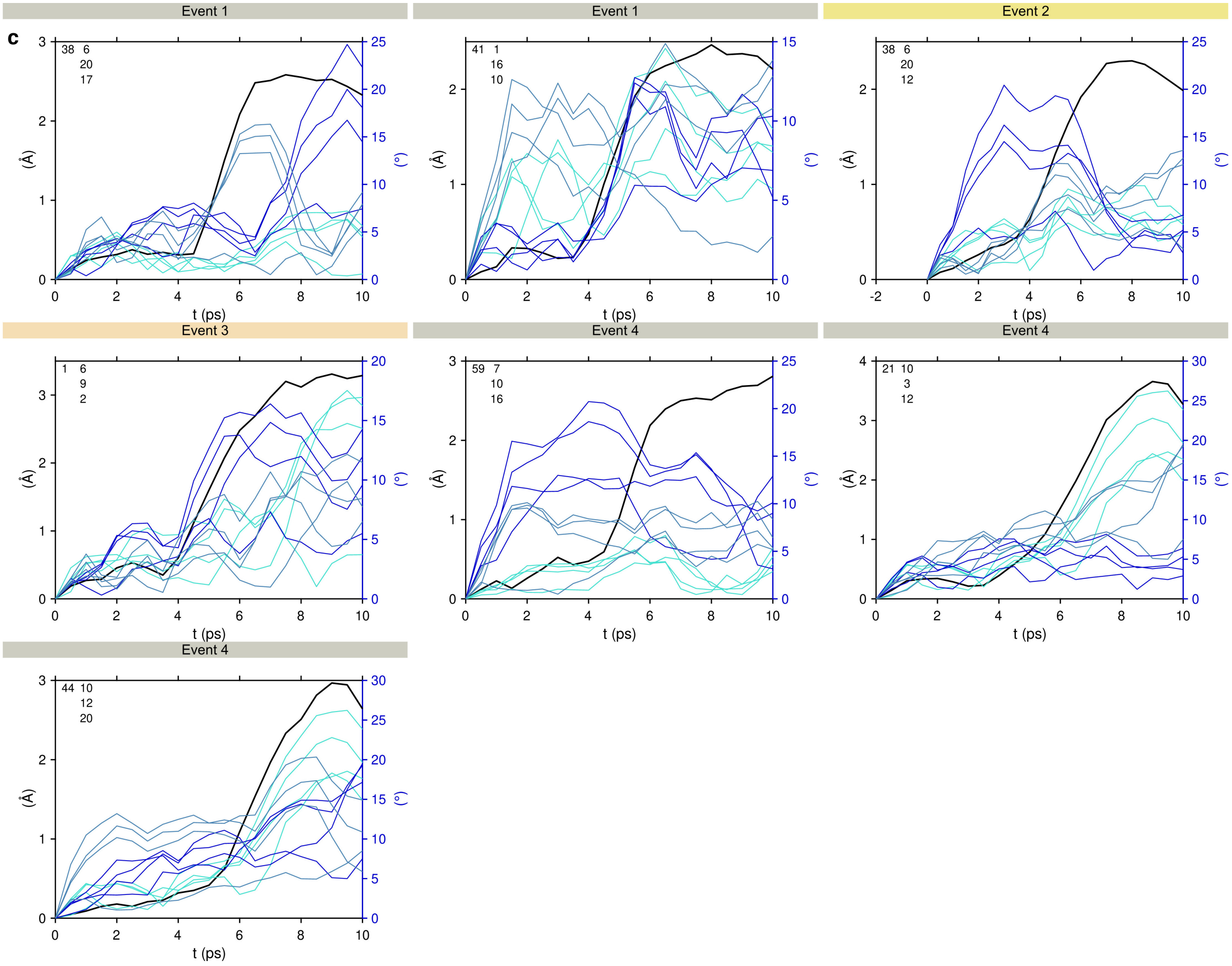

Supplementary Figure 16. Characterization of Li-ion migration events, and their correlation with rotational displacements of PS_4^{3-} anions, in LPS glass at 400 K (MD run #8). See the caption of Supplementary Fig. 8 for additional details.

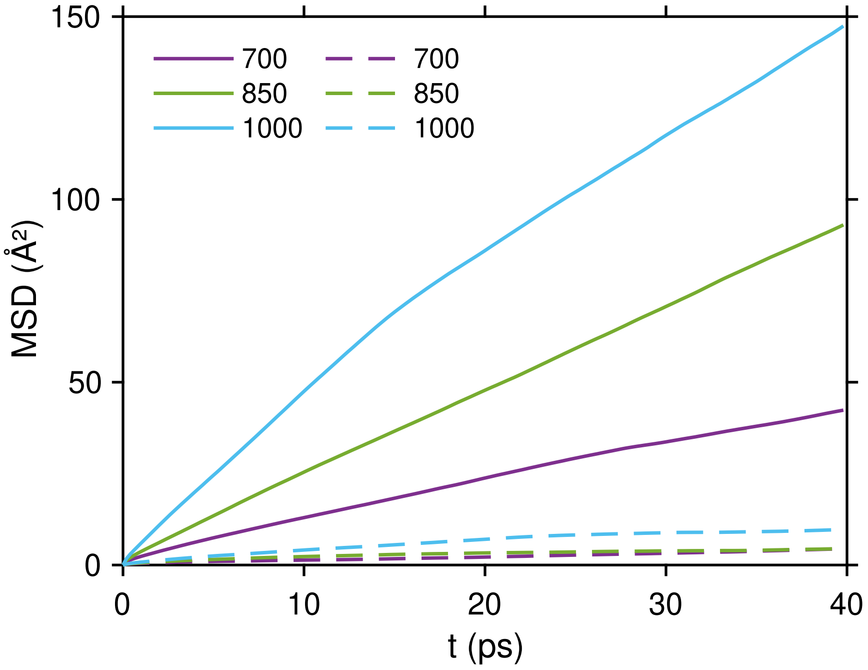

Supplementary Figure 17. Mean squared displacement of Li^+ (solid lines) and PS_4^{3-} (dashed lines).

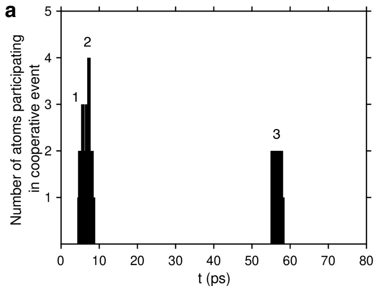

b

Event	Time	ID1	ID2	ID3	ID4
1	5.7	3	49	50	
2	7.3	3	19	50	52
3	56.7	15	37		

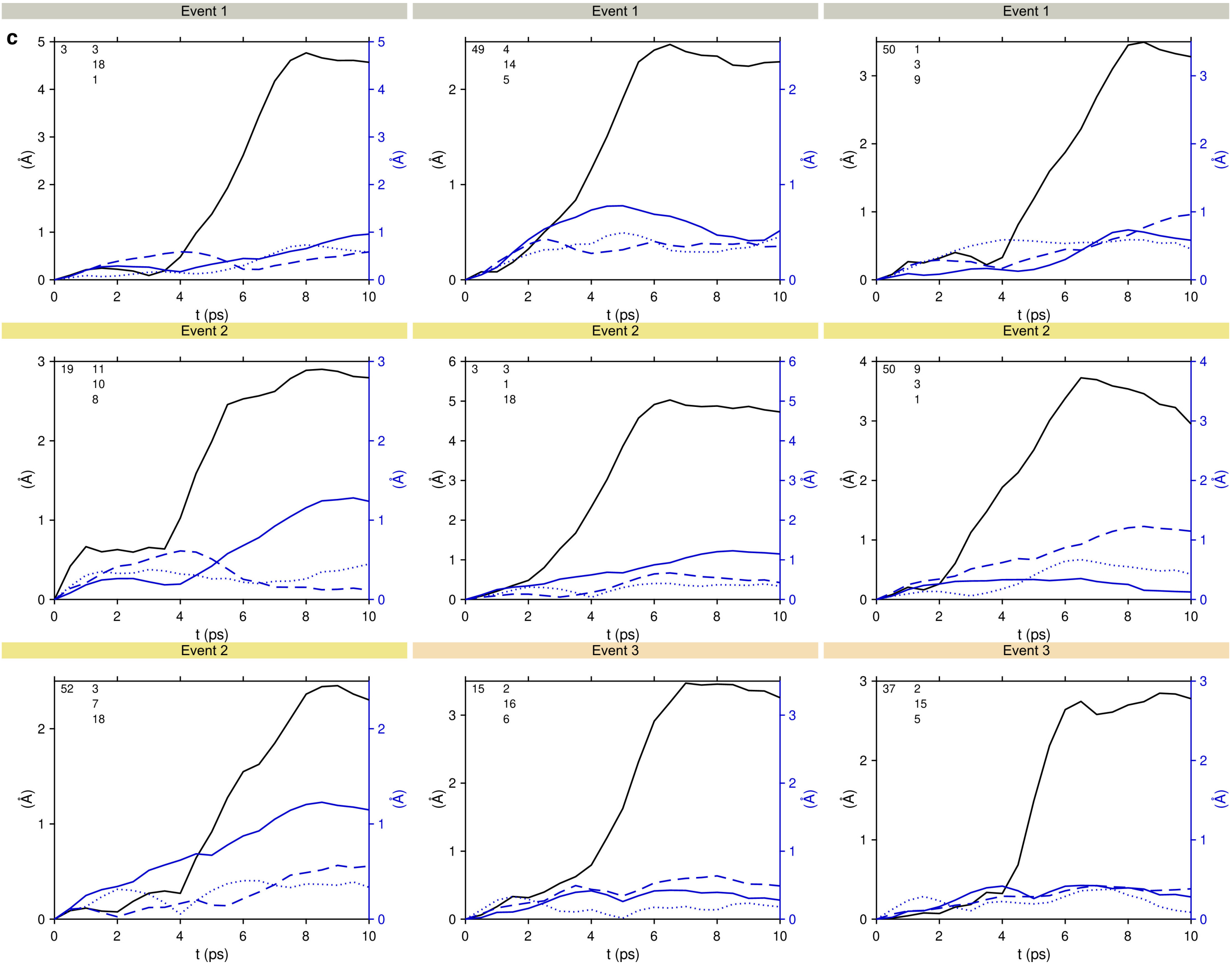

Supplementary Figure 18. Characterization of Li-ion migration events, and their correlation with displacements of PS_4^{3-} anions, in LPS glass at 300 K (MD run #1). Panels (a-b) are described in the caption of Supplementary Fig. 8. (c) The displacement of Li-ions vs. time is shown with a black curve and plotted on the left ordinate. The displacement of the 3 nearest-neighbor PS_4 anions are shown with blue curves; these data are plotted on the right ordinate.

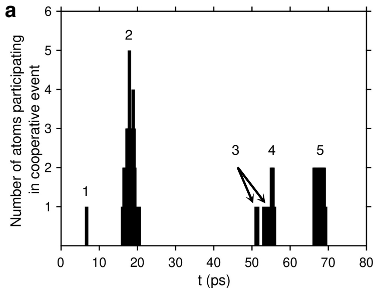

b

Event	Time	ID1	ID2	ID3	ID4	ID5
1	6.7	37				
2	17.9	1	29	39	44	116
3	51.1	57				
3	51.5	19				
4	55.3	17	47			
5	68.1	17	47			

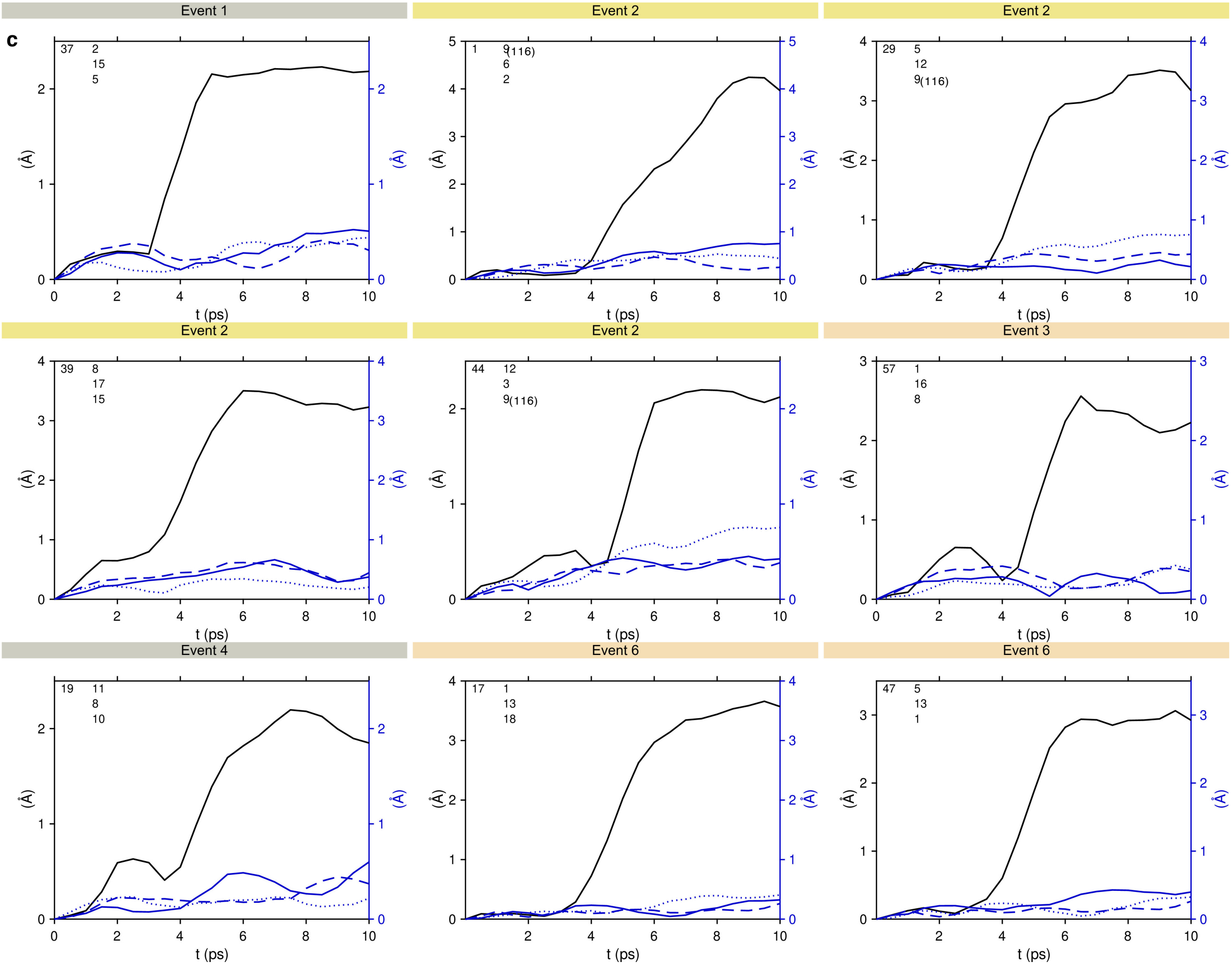

Supplementary Figure 19. Characterization of Li-ion migration events, and their correlation with displacements of PS_4^{3-} anions, in LPS glass at 300 K (MD run #2). Panels (a-b) are described in the caption of Supplementary Fig. 8. (c) The displacement of Li-ions vs. time is shown with a black curve and plotted on the left ordinate. The displacement of the 3 nearest-neighbor PS_4 anions are shown with blue curves; these data are plotted on the right ordinate.

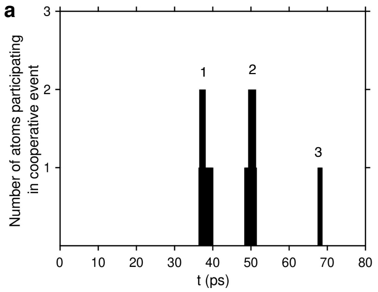

b

Event	Time	ID1	ID2
1	37.5	7	32
2	49.7	7	32
3	68.1	26	

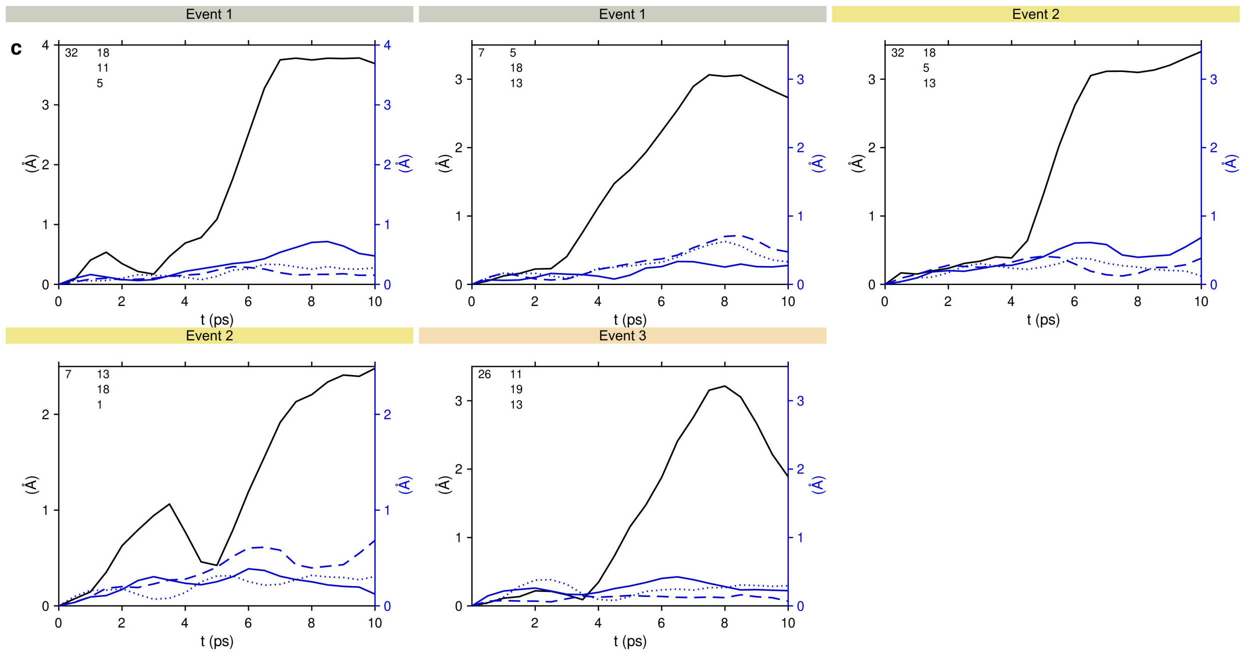

Supplementary Figure 20. Characterization of Li-ion migration events, and their correlation with displacements of PS_4^{3-} anions, in LPS glass at 300 K (MD run #3). Panels (a-b) are described in the caption of Supplementary Fig. 8. (c) The displacement of Li-ions vs. time is shown with a black curve and plotted on the left ordinate. The displacement of the 3 nearest-neighbor PS_4 anions are shown with blue curves; these data are plotted on the right ordinate.

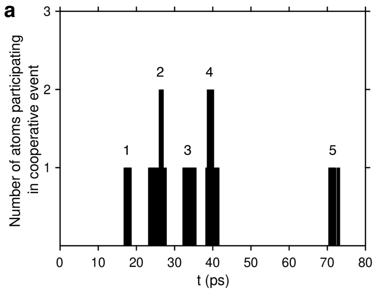

b

Event	Time	ID1	ID2
1	17.7	52	
2	26.3	19	110
3	33.9	40	
4	39.7	7	13
5	71.3	52	

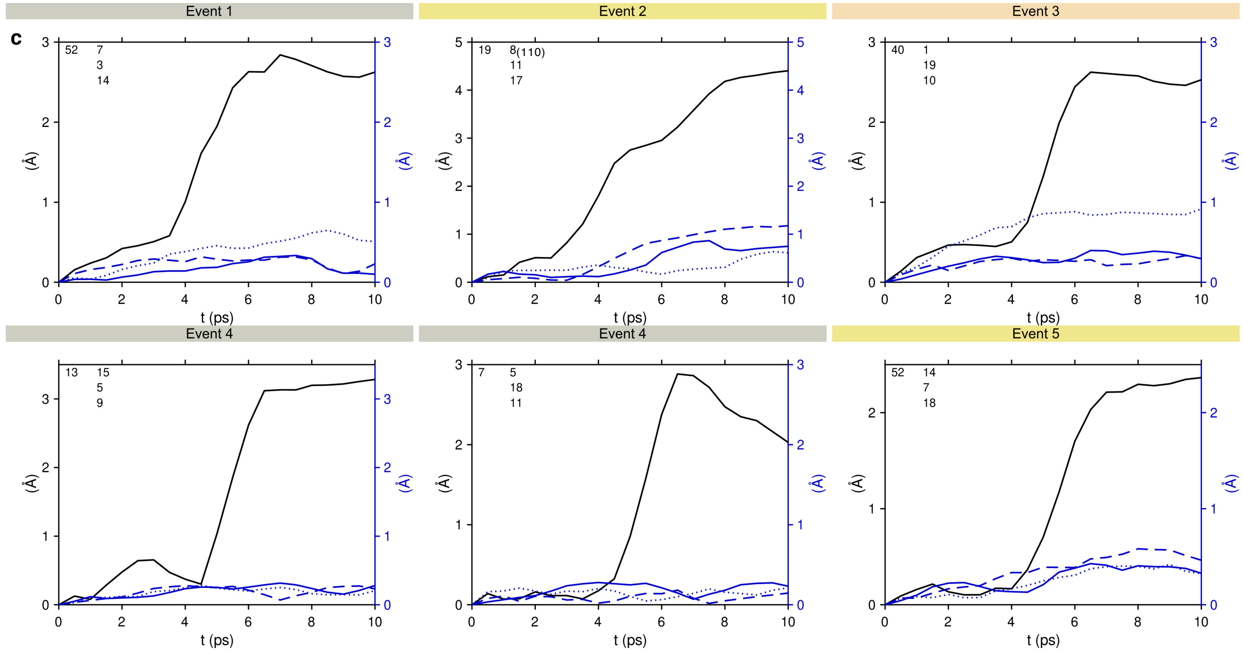

Supplementary Figure 21. Characterization of Li-ion migration events, and their correlation with displacements of PS_4^{3-} anions, in LPS glass at 300 K (MD run #4). Panels (a-b) are described in the caption of Supplementary Fig. 8. (c) The displacement of Li-ions vs. time is shown with a black curve and plotted on the left ordinate. The displacement of the 3 nearest-neighbor PS_4 anions are shown with blue curves; these data are plotted on the right ordinate.

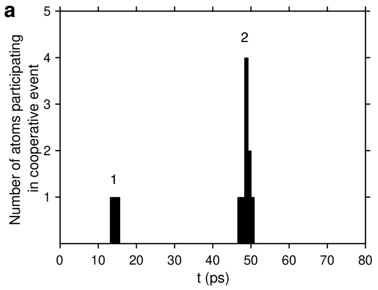

b

Event	Time	ID1	ID2	ID3	ID4
1	14.5	14			
2	48.7	14	32	39	59

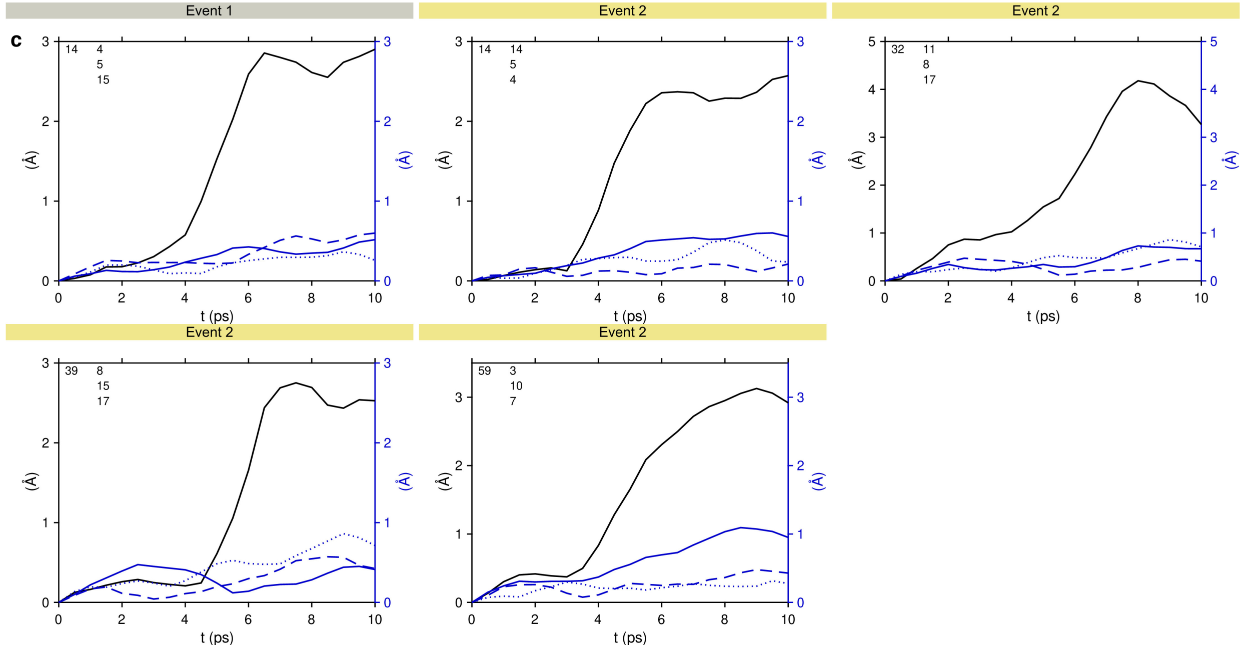

Supplementary Figure 22. Characterization of Li-ion migration events, and their correlation with displacements of PS_4^{3-} anions, in LPS glass at 300 K (MD run #5). Panels (a-b) are described in the caption of Supplementary Fig. 8. (c) The displacement of Li-ions vs. time is shown with a black curve and plotted on the left ordinate. The displacement of the 3 nearest-neighbor PS_4 anions are shown with blue curves; these data are plotted on the right ordinate.

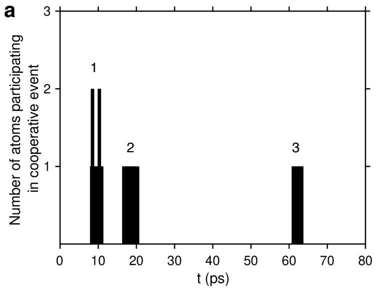

b

Event	Time	ID1	ID2
1	8.5	8	30
1	10.3	8	30
2	17.9	8	
3	62.1	13	

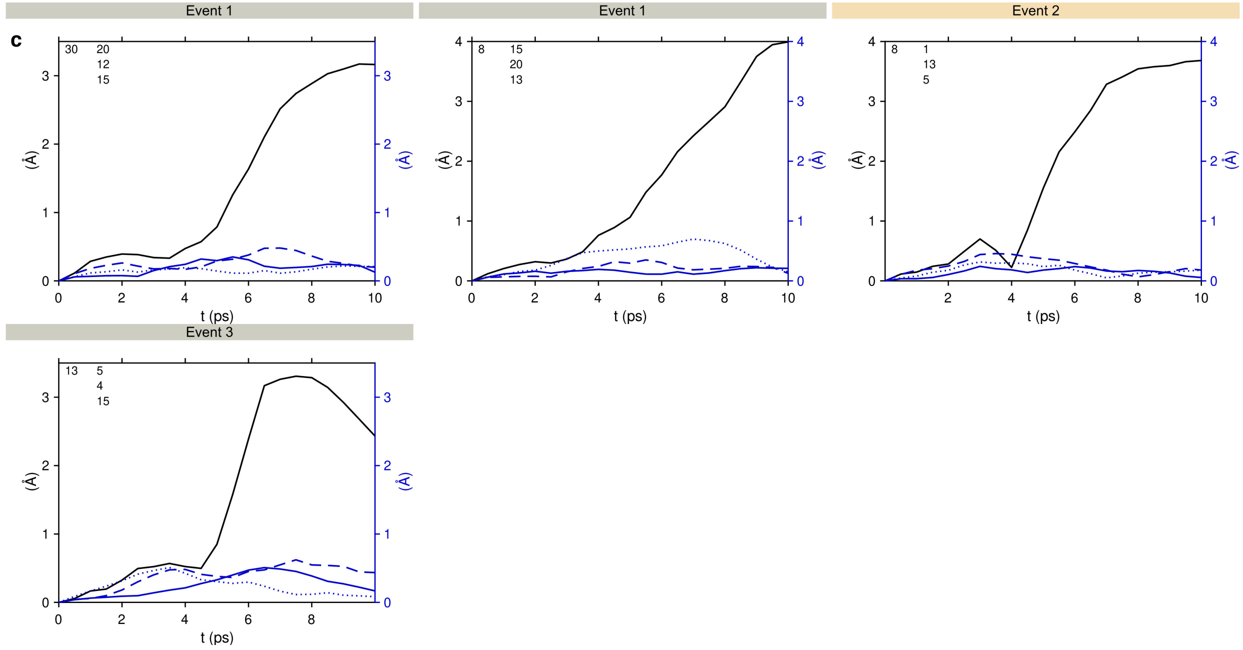

Supplementary Figure 23. Characterization of Li-ion migration events, and their correlation with displacements of PS_4^{3-} anions, in LPS glass at 300 K (MD run #6). Panels (a-b) are described in the caption of Supplementary Fig. 8. (c) The displacement of Li-ions vs. time is shown with a black curve and plotted on the left ordinate. The displacement of the 3 nearest-neighbor PS_4 anions are shown with blue curves; these data are plotted on the right ordinate.

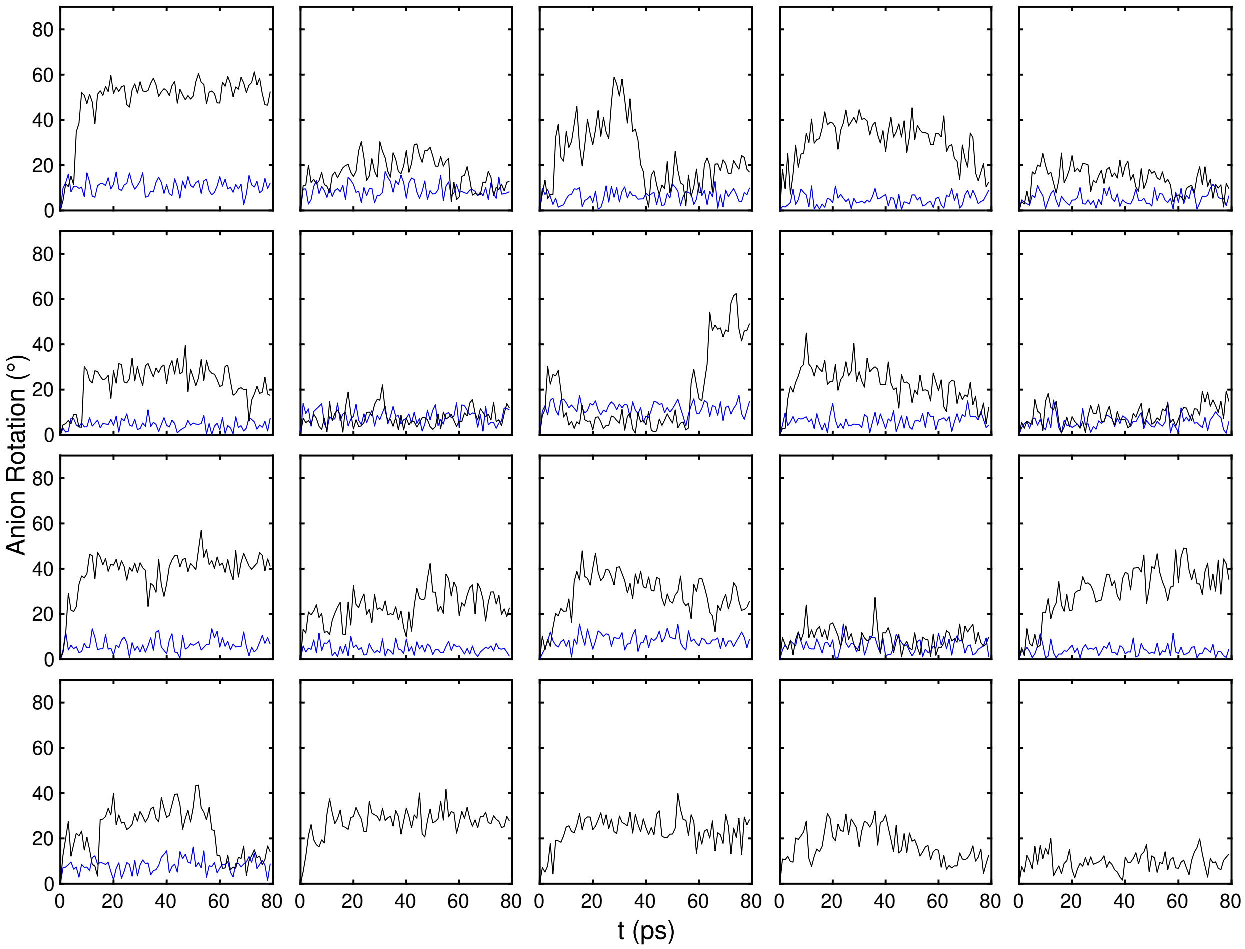

Supplementary Figure 24. Rotational displacements of individual PS_4 anions in glassy Li_3PS_4 (black) and in crystalline γ-Li_3PS_4 (blue) as a function of MD time at 300 K and 1 bar. The γ-Li_3PS_4 crystal structure is from Homma *et al.*[3]

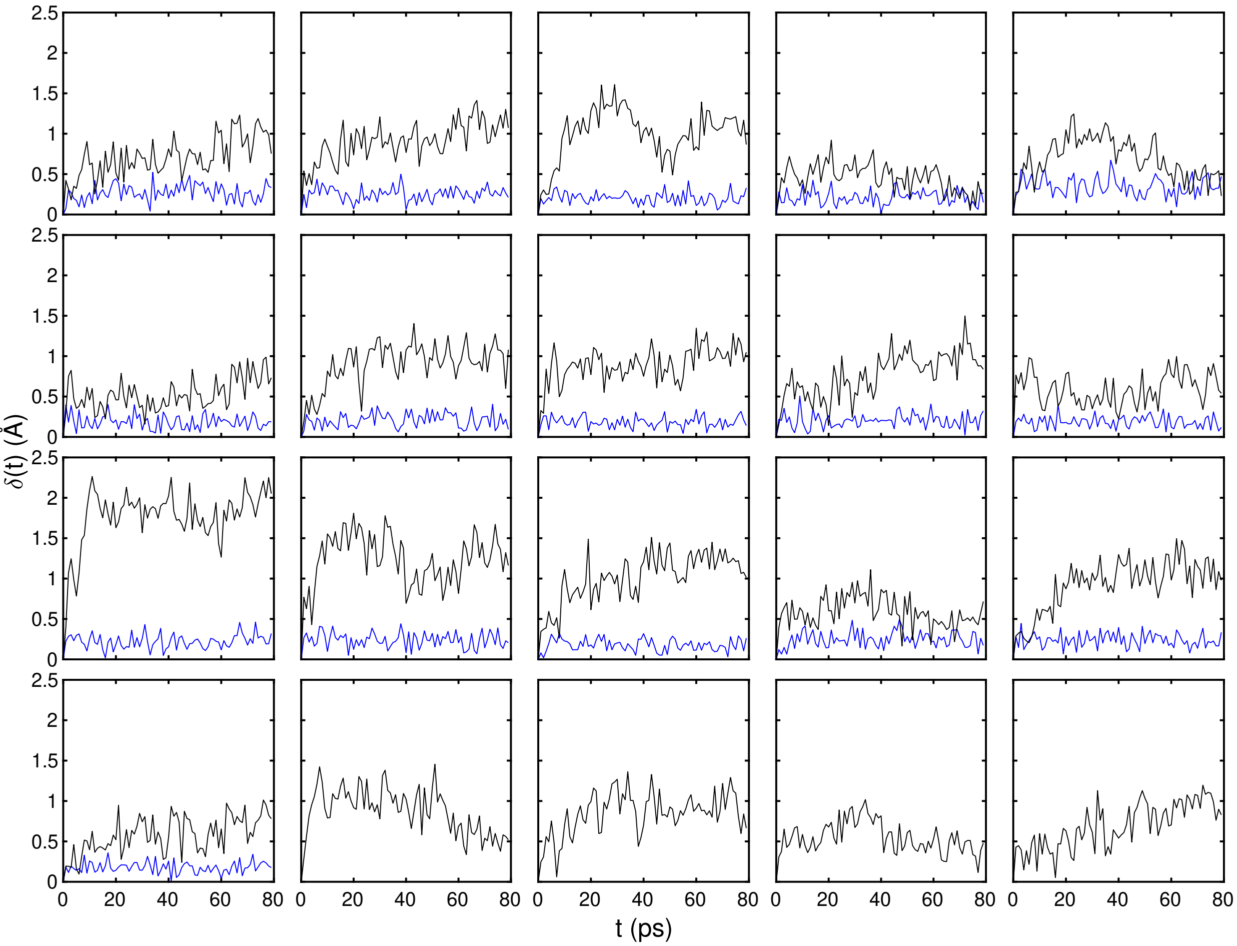

Supplementary Figure 25. Translational displacements of individual anions in glassy Li_3PS_4 (black) and in crystalline γ-Li_3PS_4 (blue) as a function of MD time at 300 K and 1 bar. The γ-Li_3PS_4 crystal structure is from Homma *et al.*[3]

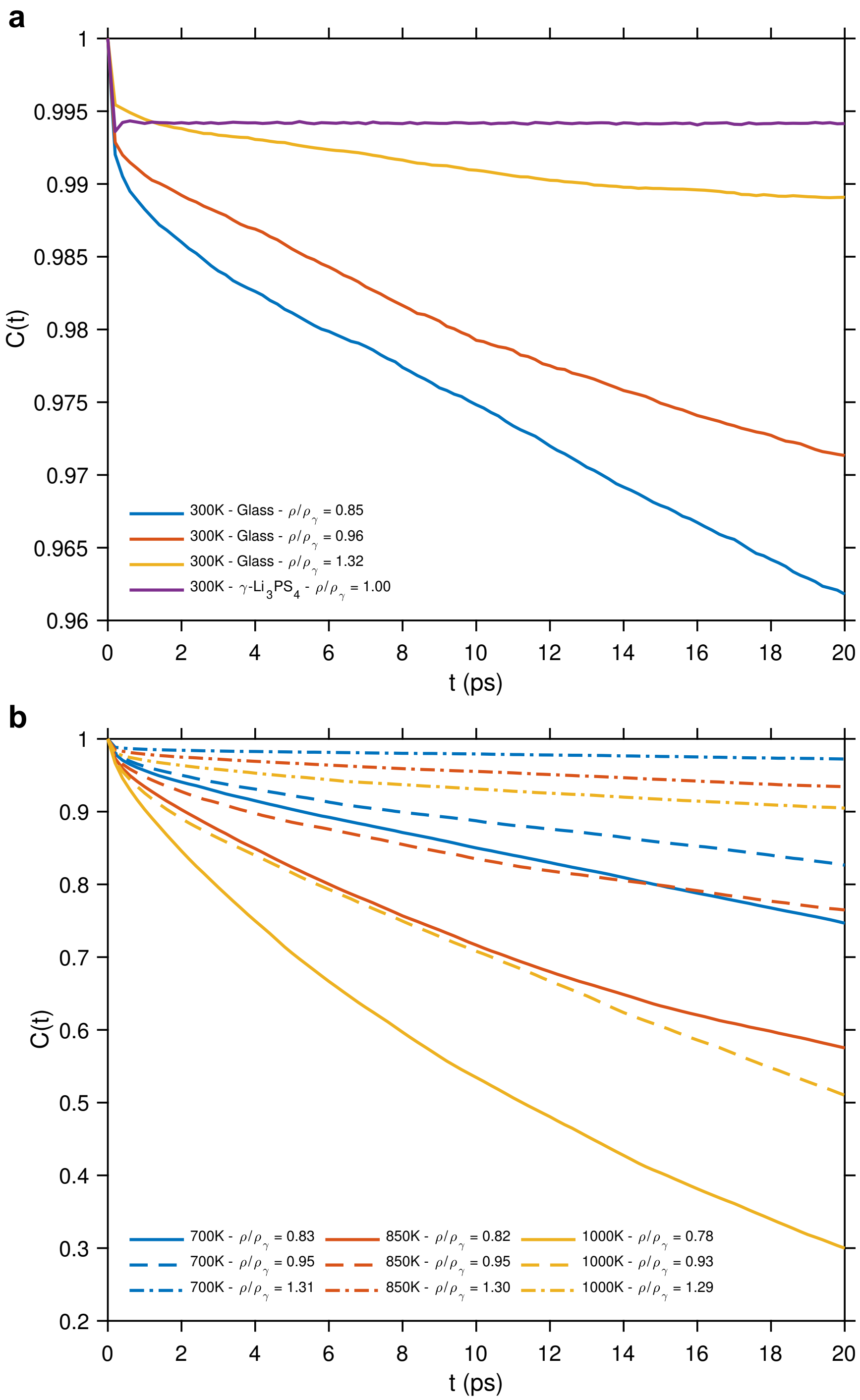

Supplementary Figure 26. PS_4 anion reorientation time-correlation function, $C(t) = \langle \boldsymbol{u}(t+t') \cdot \boldsymbol{u}(t') \rangle$, as a function of volume (density) and temperature. Here, $\boldsymbol{u}$ is a unit vector defined from the anion center of mass (P atom) to covalently-bonded S atoms. ρ is the average glass density, and ρ_γ is the density of crystalline γ-Li_3PS_4 at 300 K and 1 bar. (a) Comparison of glassy and crystalline γ-Li_3PS_4 at 300 K. (b) Behavior of the glass at 700, 850, and 1000 K.

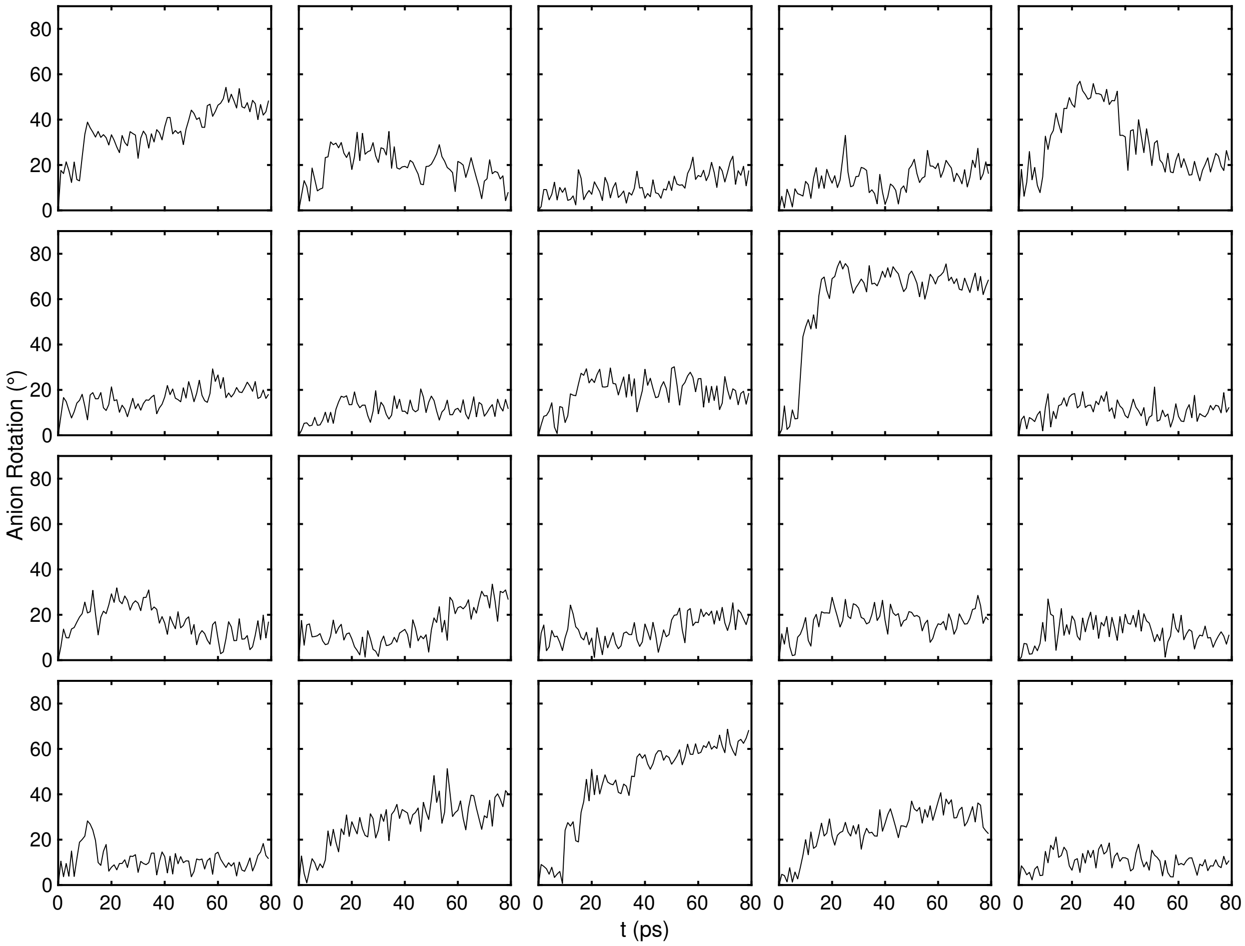

Supplementary Figure 27. Rotational displacements of individual PS_4 anions in glassy Li_3PS_4 as a function of MD time at 300 K and an applied hydrostatic pressure of 1 GPa. The applied pressure results in a density of 1.76 g cm^{-3}.

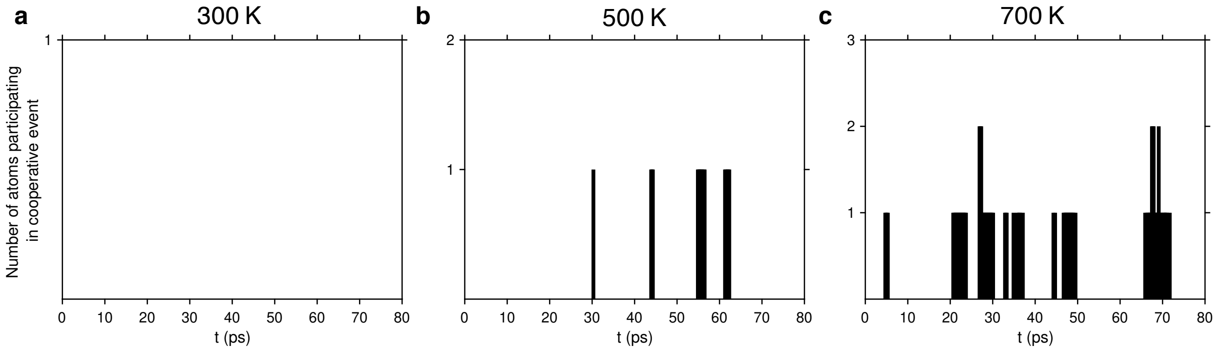

Supplementary Figure 28. Li-ion migration events (identified using Eq. 3) during AIMD simulations at (a) 300 K & 1 bar, (b) 500 K and 1 bar, and (c) 700 K and 1 bar in which the anion degrees of freedom were frozen. A similar analysis is shown in Fig. S6 (for 500 and 700 K) for the case where the PS_4 dynamics were not constrained.

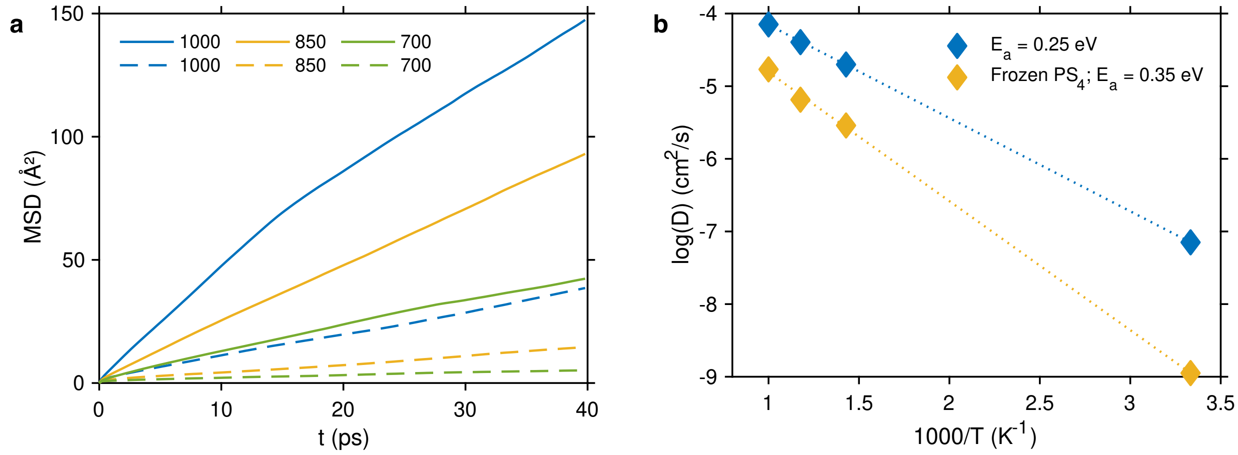

Supplementary Figure 29. Calculated Li-ion diffusion data. (a) Mean squared displacement of Li-ions in the presence of frozen PS_4 anions (dashed lines) and without constraints on the PS_4 dynamics (solid lines). (b) Diffusion coefficients with frozen anions (yellow diamonds) and without constraints on the anions (blue diamonds). The dotted lines in panel (b) depict a linear fit to the high temperature diffusivity data, which is extrapolated to 300 K.

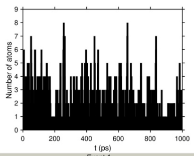

Event	Time	ID1	ID2	ID3	ID4	ID5	ID6	ID7	ID8
1	6.3	9	529	1030	1352	3817	3850		
2	51.3	34	400	837	842	1248	1269	1595	
3	256.1	46	566	847	901	1090	1117	1458	1506
4	259.1	469	627	834	1066	1093	1458	4074	
5	262.7	50	419	494	1400	1415	3602	3656	
6	371.7	460	802	2726	2754	2807			
7	389.1	388	532	611	918	1401			
8	656.3	30	75	1380	1791	2379	2406	2817	2844

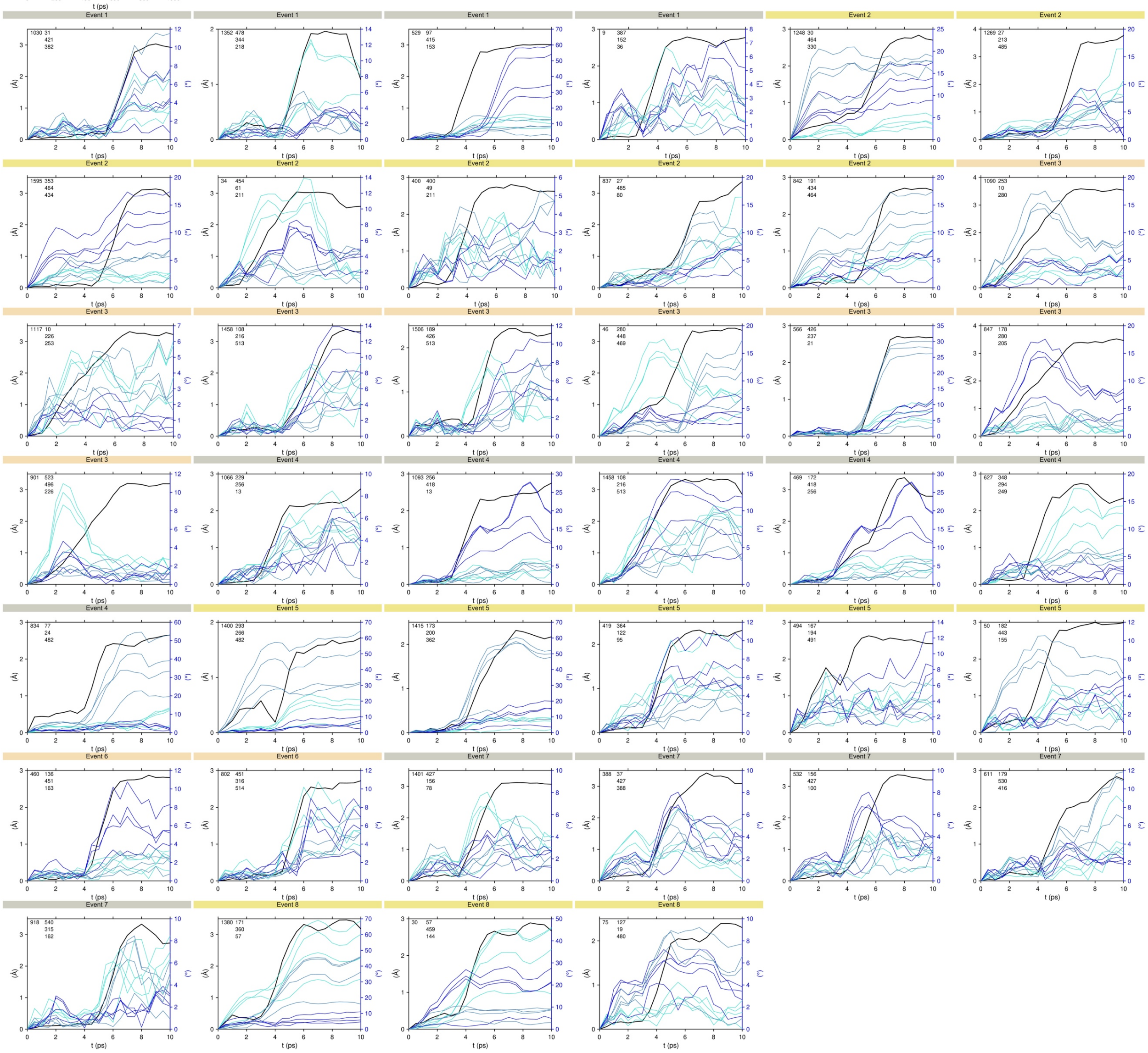

Supplementary Figure 30. Characterization of Li-ion migration events, and their correlation with rotational displacements of PS_4^{3-} anions, in LPS glass at 300 K using a classical interatomic potential from Supplementary Ref .[5] These simulations used a simulation cell containing 4,320 atoms, corresponding to a system size 27 times larger than that used for AIMD. These data confirm that the paddlewheel mechanism also is present in the classical simulation, supporting the conclusions reached with AIMD simulations, and ruling out finite-size effects.

Supplementary Tables

Supplementary Table 1. Density (in units of g cm^{-3}) of 75Li_2S-25P_2S_5 glass, and crystalline γ-Li_3PS_4 and β-Li_3PS_4 as a function of pressure averaged over 80 ps. The calculated instantaneous density of 75Li_2S-25P_2S_5 as a function of hydrostatic pressure is shown in Supplementary Figure 2. The experimental density of 75Li_2S-25P_2S_5 glasses prepared at room temperature with a pressure of 1.8 and 3.6 kBar were reported as 1.45 and 1.68 g cm^{-3}, respectively.[1] Baba and Kawamura calculated an AIMD density of 1.79 g cm^{-3} using a computational cell that was constrained to be cubic.[2] For comparison, the experimental density of γ-Li_3PS_4 (1.93 g cm^{-3})[3] is also given.

System	1 bar	3.6 kbar	10 kbar	100 kbar
Glass (Calc.)	1.56	1.65	1.76	2.42
γ-Li_3PS_4 (Calculated, 300 K)	1.83	–	–	–
γ-Li_3PS_4 (Experiment, 297 K)[3]	1.93	–	–	–
β-Li_3PS_4 (Calculated, 300 K)	1.76			
β-Li_3PS_4 (Experiment, 637 K)[3]	1.85			

Supplementary References

1. Sakuda, A., Hayashi, A., Takigawa, Y., Higashi, K. & Tatsumisago, M. Evaluation of elastic modulus of Li_2S–P_2S_5 glassy solid electrolyte by ultrasonic sound velocity measurement and compression test. *J. Ceram. Soc. Japan* **121**, 946–949 (2013).
2. Baba, T. & Kawamura, Y. Structure and Ionic Conductivity of Li_2S–P_2S_5 Glass Electrolytes Simulated with First-Principles Molecular Dynamics. *Front. Energy Res.* **4**, 1–10 (2016).
3. Homma, K. *et al.* Crystal structure and phase transitions of the lithium ionic conductor Li_3PS_4. *Solid State Ionics* **182**, 53–58 (2011).
4. Ohara, K. *et al.* Structural and electronic features of binary Li_2S-P_2S_5 glasses. *Sci. Rep.* **6**, 21302 (2016).
5. Kim, J.-S. *et al.* Atomistic Assessments of Lithium-Ion Conduction Behavior in Glass–Ceramic Lithium Thiophosphates. *ACS Appl. Mater. Interfaces* **11**, 13–18 (2018).
